# Excessive use of medically important antimicrobials in food animals in Pakistan: a five-year surveillance survey

**DOI:** 10.1080/16549716.2019.1697541

**Published:** 2019-12-04

**Authors:** Mashkoor Mohsin, Thomas P. Van Boeckel, Muhammad Kashif Saleemi, Muhammad Umair, Muhammad Noman Naseem, Cheng He, Ahrar Khan, Ramanan Laxminarayan

**Affiliations:** a Institute of Microbiology, University of Agriculture, Faisalabad, Pakistan; b Institute for Environmental Decisions, ETH Zurich, Zurich, Switzerland; c Center for Disease Dynamics, Economics & Policy, Washington, DC, USA; d Department of Pathology, University of Agriculture, Faisalabad, Pakistan; e College of Veterinary Medicine, China Agricultural University, Beijing, China; f Shandong Vocational Animal Science and Veterinary College, Weifang, China; g Princeton Environment Institute, Princeton, NJ, USA

**Keywords:** Antimicrobial Resistance, Antimicrobial consumption, poultry, LMICs, Pakistan, colistin, surveillance

## Abstract

Demand for poultry meat is rising in low- and middle-countries, driving the expansion of large commercial farms where antimicrobials are used as surrogates for hygiene, good nutrition. This routine use of antimicrobials in animal production facilitates the emergence and spread of antibiotic-resistant pathogens. Despite potentially serious consequences for the animal industry, few studies have documented trends in antimicrobial use (AMU) at the farm-level in low- and middle-income countries. The objective of this study was to estimate AMU in a broiler chicken farm in Pakistan over a five-year period and to extrapolate national AMU in commercial broiler farming. Between 2013 and 2017, we monitored AMU in 30 flocks from a commercial broiler farm in Punjab, the most populous province of Pakistan. The amount of antimicrobials administered was calculated in milligram/population unit of the final flock weight (mg/fPU) and in used daily dose (UDD). The annual on-farm antimicrobial use was 250.84 mg of active ingredient per kilogram of the final flock weight. This consumption intensity exceeds the amount of antimicrobial used per kilogram of chicken of all countries in the world except China. Measured in mg per kg of final flock weight or population unit (fPU), medically important drugs such as colistin (31.39 mg/fPU), tylosin (41.71 mg/fPU), doxycycline (81.81 mg/fPU), and enrofloxacin (26.19 mg/fPU) were the most frequently used antimicrobials for prophylactic or therapeutic use. Lincomycin was the most frequently used antimicrobial used in-feed (29.09 mg/fPU). Our findings suggest that the annual consumption of antimicrobials in the broiler sector in Pakistan could be as high as 568 tons. This alarmingly high consumption estimate is the first baseline study on antimicrobial use in animals in Pakistan. Our findings call for immediate actions to reduce antimicrobial use in Pakistan, and countries with comparable farming practices.

## Background

The rise of antimicrobial resistance (AMR) is a global phenomenon driven by antimicrobial use (AMU). Global data suggest that AMU in food animals far outweighs its consumption in human medicine [1]. Mounting evidence suggests that the overuse of antimicrobials in food animals contributes to the emergence of drug-resistant bacterial infections in animals and humans [2]. Several antimicrobials used in veterinary medicine are also on the World Health Organization (WHO) list of critically important antimicrobials for human medicine [3]. Surveillance of AMU in animals is already monitored in some high-income countries; however, in the low- and middle-income countries (LMICs) monitoring systems remain largely absent [4]. Although the estimation of AMU in food animals is one of the key objectives of the Global Action Plan on AMR WHO, there are few national-level estimates of veterinary antimicrobial use in LMICs. Pakistan has recently drafted its National Action Plan in 2017 in response to the WHO’s Global Action Plans on AMR, and committed to addressing the AMR regulatory and policy issue according to the “One Health” approach [5]. This calls for monitoring AMU in livestock and poultry to fill an important knowledge gap. This study was designed to study AMU patterns both as prophylactic or therapeutic and in-feed from a representative commercial broiler farm between 2013 and 2017.

## Methods

We monitored antimicrobial consumption for a five-year period (2013–2017) on a commercial broiler farm located in Faisalabad, Pakistan. Overall, AMU records of 30 flocks (F_1_–F_30_), consisted of 595,300 birds, were monitored over a period of five years. Standard feeding protocols using starter, grower, and finisher rations were followed. Antimicrobials were administered for prophylactic (enrofloxacin and colistin at days 1–4, tylosin and doxycycline at days 20–24) or treatment purposes only through water, while the antimicrobials used as feed additive came premixed from the feed manufacturer. Farm records were maintained to monitor antimicrobial use, including brands and quantities used. Records for the production output in terms of final flock weight were also maintained (Table S1).

### Population unit

In a broiler production system, if the entire flock is considered a single biomass with initial weight regarded when the chicks enter the system and final weight when the production is complete, flock-biomass can be calculated in Population Unit (PU) with initial and final weights termed as iPU and fPU, respectively. The antimicrobial consumption per population unit can be expressed in milligrams with respect to fPU:

where *F*_FW kg_ is the final flock weight in kg.

### AMU per fPU calculation

Total AMU in mg for 30 flocks was calculated by the addition of antimicrobials used as prophylactic (*U*_P mg_), treatment (*U*_T mg_) and feed additive, latter calculated by multiplying the total feed consumed in kg (*C*_F kg_) with the inclusion rate of antimicrobial in feed (*IR*_mg/kg_) obtained from manufacturer. The division of total AMU with the sum of corresponding final flock weights (*F*_FW kg_) gives the total consumption per fPU.

### Used daily dose calculation

Daily antimicrobial consumption during prophylactic or treatment courses was calculated by dividing flock water consumption in liters (*C*_W L_) times the antimicrobial inclusion rate per liter of water (*IR*_mg/L_) by the flock weight (*F*_W kg_) at respective day (*d*). Used daily dose (UDD) was calculated as the average of daily antimicrobial consumption in corresponding prophylactic or treatment courses consisting of days (D).

The European Medicines Agency’s European Surveillance of Veterinary Antimicrobial Consumption (ESVAC) has outlined defined daily doses (DDD) values for animals in Europe. These values have been assigned according to the declared strength (content) given in the label/name or summaries of product characteristics from different countries in Europe [6]. UDD/DDD was calculated to account for the differences between used dose in our study and defined dose of ESVAC (Table 1).

**Table 1.** Total amount of antimicrobial agents used for prophylactic and treatment purposes in surveyed flocks during 2013–17

Drug class	Drug name	Flocks treated	Treated fPU kg	Used active ingredient kg	mg/fPU	UDD mg/kg	DDD mg/kg	UDD/DDD
Polymyxins	Colistin	30	1,233,404	38.72	31.39	70.54	5.1	13.83
Macrolides	Tylosin	30	1,233,404	51.45	41.71	19.21	81	0.24
	Erythromycin	1	43,101	2	46.4	30.68		
Fluoroquinolones	Enrofloxacin	30	1,233,404	32.3	26.19	50.61	10	5.06
	Norfloxacin	1	31,353	2	63.79	36.52		
Tetracyclines	Doxycycline	30	1,233,404	100.9	81.81	38.42	15	2.56
	Chlortetracycline	18	749,501	8	10.67	93.97	30	3.13
	Oxytetracycline	4	166,857	6.25	37.46	29.37	39	0.75
Aminoglycosides	Neomycin	19	792,501	9.78	12.34	31.71	24	1.32
	Streptomycin	1	42,329	0.9	21.26	9.91		
Nitrofurans	Furaltadone	19	792,602	8	10.09	70.47		
Antiviral	Amantadine	24	980,921	14.21	14.49	7.68		
Penicillin	Penicillin	1	42,329	0.3	7.09	3.3		
Polypeptides	Bacitracin	1	42,329	1.3	30.71	14.31		
Sulfonamides	Sulfamethoxypyridazine	1	38,127	0.6	15.74	15.34	23	0.67
	Sulfamethazine	1	38,127	0.6	15.74	15.34		0
Trimethoprim	Trimethoprim	1	38,127	0.3	7.87	9.2	6.4	1.44
Lincosamides	Lincomycin^a^	24	987,877	28.74	29.09	2.68	8.6	0.31
Polypeptides	Enramycin F^a^	6	245,527	3.25	13.24	1.22		
	Total	30	1,233,404	309.6	251.01			

^a^Antimicrobials premixed with feed as feed additive with an inclusion rate of 17.6 g/ton and 8 g/ton, respectively.

### Country’s AMU estimation

We used the average estimate of broilers produced per year (calculated by considering 3% mortality factor generally observed at farm) to estimate the annual antimicrobial consumption in Pakistan’s broiler industry. The approximation was made by relating average AMU (kg) over estimated number of broilers produced per year at studied farm with the number of commercial broilers produced in the country in the year 2017–18 according to Pakistan Economic Survey 2017–18 [7].

## Results

A total of 17 antimicrobials (belonging to 11 classes) were used for therapeutic or prophylactic purposes, among 30 flocks over a period of five years. In addition, two antimicrobials (lincomycin or enramycin) were used in-feed (Table 1). Among the antimicrobials used for therapeutic or prophylactic purposes, colistin, tylosin, enrofloxacin, and doxycycline were the most frequently used antimicrobials (each 100%, 30/30 flocks) followed by amantadine (80%, 24/30), neomycin and furaltadone (each 63%, 19/30), and chlortetracycline (60%, 18/30) (Table 1). The most commonly used antimicrobials for therapeutic or prophylactic purposes measured by mg/fPU consisted of doxycycline (81.81 mg/fPU), tylosin (41.71 mg/fPU), and colistin (31.39 mg/fPU). When using the UDD metric, the top three antimicrobials used were chlortetracycline (93.97 mg/kg), colistin (70.54 mg/kg), and furaltadone (70.47 mg/kg). In addition, lincomycin was the most commonly used antimicrobial agent as a feed additive (29.09 mg/fPU) followed by enramycin (13.23 mg/fPU) (Table 1). Lincomycin was consumed every year in-feed except in 2014, where lincomycin was replaced by enramycin.

Between 2013 and 2017, the total AMU was 309.6 in kg and 251.01 in mg/fPU while the average annual use was found to be 61.92 in kg and 250.84 in mg/fPU (Table 1 and Table S3). AMU as feed additive constituted a share of 10.33% in kg and mg/fPU in total AMU. Among the 23 products used for prophylactic or treatment purpose, 52% (12/23) contained colistin followed by enrofloxacin (35%, 8/23), tylosin (30%, 7/23), doxycycline and amantadine (26%, 6/23 each) (Table S2).

**Figure 1.**
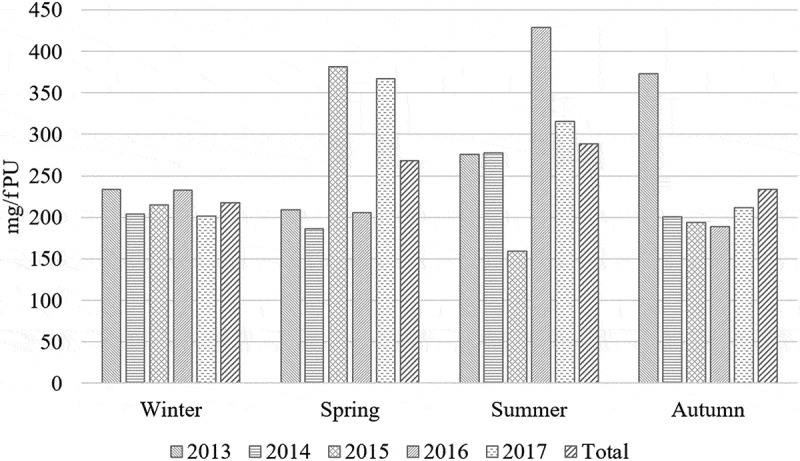
Seasonal effect on AMU in 30 flocks during 2013–17

Seasonal variation carries a non-negligible effect on AMU with an increase of 24.79% in total mg/fPU in summers compared to winters (Figure 1). With an average of 115,488 broilers produced (accounting 3% mortality) and AMU of 62 kg per year in the present study, 1,057.65 million broilers produced in Pakistan in the year 2017–18 would have consumed ≈ 568 tons of antimicrobial [7].

## Discussion

The poultry sector in Pakistan is producing 1,057.65 million broilers annually and is the second largest export industry in value after textiles [7]. In this study, the average annual on-farm antimicrobial use was 250.84 mg/kg of the final flock weight. This consumption intensity exceeds the amount of antimicrobial used per kilogram of chicken of all countries in the world except China [8]. Interestingly, average AMU (250.84 mg/fPU) in the present study is substantially higher than the estimated global use (≈ 148 mg/PCU) in chicken [1]. In particular, the use of colistin vastly exceeds the DDD values of colistin for broilers outlined by the ESVAC. Colistin, enrofloxacin and chlortetracycline were 13.8, 5 and 3.1 times higher than their respective DDD values (Table 1), respectively [6]. From 19 different antimicrobials, seven (colistin, tylosin, erythromycin, enrofloxacin, norfloxacin, neomycin, and streptomycin) are categorized as critically important for human medicine by WHO [3]. Colistin, considered as the last-resort antibiotic for the treatment of multidrug-resistant infections in human, was the most frequent (100%) antimicrobial used with an average of 7.74 kg per year and 31.39 mg/fPU. Compared to the colistin use reports from Vietnam (5.3 and 1.8 mg/kg) [9] and Morocco (8.4 mg/kg) [10], our study reveals use at threatening levels (31.39 mg/fPU). The plasmid-mediated mobile colistin resistance *mcr-1* gene represents a new threat due to the transferability of this gene between bacteria. After the discovery of *mcr-1* from broilers and human in Pakistan [11,12], such a high level use of colistin poses a major threat of selecting resistant microorganism, as reported in China [13]. In the present study, in-feed AMU shares 10.33% in total AMU, raising questions for policy makers to enforce restrictions on in-feed antimicrobial use in Pakistan. Although the present study was carried out in a single farm in Punjab, one of its notable strengths is the long follow-up period [9,14]. Our study has also limitations in terms of extrapolation for annual country’s AMU in intensive commercial broiler sector based on one commercial broiler production unit. Nevertheless, we also believe this farm to be generally representative of ongoing broiler farming practices in Pakistan. These extrapolations were based on two assumptions: first because the surveyed farm is located in Punjab province which contributes over 70% of the country’s broiler production [15]. Second that the flock size across commercial broiler enterprises in Punjab ranged between 20,000 and 38,000 birds [16], thus our farm size (20,000) corresponds to average farm size likely leading to similar farming practices. Based on this cautionary extrapolation, we estimated annual AMU of Pakistan in commercial broiler industry could be around 568 tons. Previous studies based on extrapolation from country sales data had estimated AMU for all animal species to be at 1,031 tons in 2013 [17].

## Conclusion

Our findings emphasize the urgent need to phase out the use of critically important antimicrobials in chicken in Pakistan to prevent a public health crisis. The trends reported in our study highlight the fact that antimicrobials are being largely overused in broiler production in Pakistan, calling for urgent action.

## Supplementary Material

Supplemental Material

Supplemental Material

Supplemental Material

## Paper context

Surveillance of antimicrobial use in food animals is one of the key objectives of Global and National Action Plan on antimicrobial resistance. However, no published studies exist on AMU in poultry in Pakistan. The article described a five-year surveillance data of AMU in poultry from Pakistan. This pilot data are of immediate importance for policy makers in Pakistan and countries with comparable farming practices to avoid a public health crisis.
