# Supplementary material for: Excessive use of medically important antimicrobials in food animals in Pakistan: a five-year surveillance survey
Source: Glob Health Action. 2019 Dec 4;12(Suppl):1697541. doi: 10.1080/16549716.2019.1697541 (PMC6896466; doi:10.1080/16549716.2019.1697541)
Supplement: Supplemental Material [file ZGHA_A_1697541_SM6956.pdf]

Table S3: Total amount of antimicrobial used in each year and average annual use

		2013		2014		2015		2016		2017			
Drug Count	Drug Name	T kg	mg/fPU	T kg	mg/fPU	T kg	mg/fPU	T kg	mg/fPU	T kg	mg/fPU	Avg kg	Avg mg/fPU
1	Colistin	12.8	50.63	6.08	24.77	3.48	14.42	6.68	27.84	9.68	38.14	7.74	31.16
2	Tylosin	11.6	45.89	8.7	35.43	10.35	42.88	10.7	44.6	10.1	39.79	10.29	41.72
3	Erythromycin	0	0	0	0	0	0	2	46.4	0	0	2	46.4
4	Enrofloxacin	5.8	22.94	4.7	19.14	5.1	21.13	7	29.18	9.7	38.22	6.46	26.12
5	Norfloxacin	0	0	0	0	0	0	2	63.79	0	0	2	63.79
6	Doxycycline	23.2	91.77	17.4	70.87	20.7	85.77	19.4	80.87	20.2	79.58	20.18	81.77
7	Chlortetracycline	1.4	10.98	1.8	14.53	1.4	11.82	1.2	9.53	2.2	8.67	1.6	11.11
8	Oxytetracycline	1.5	34.88	3.5	42.21	0	0	1.25	30.54	0	0	2.08	35.88
9	Neomycin	2.22	13.02	4.62	37.3	0.42	3.55	1.86	14.77	0.66	2.6	1.96	14.25
10	Streptomycin	0	0	0.9	21.26	0	0	0	0	0	0	0.9	21.26
11	Furaltadone	1.05	8.24	1.35	10.9	1.05	8.87	2.9	17.16	1.65	6.5	1.6	10.33
12	Amantadine	0.76	6.11	1.29	7.93	3.32	16.58	4.8	20.01	4.04	15.92	2.84	13.31
13	Penicillin	0	0	0.3	7.09	0	0	0	0	0	0	0.3	7.09
14	Bacitracin	0	0	1.3	30.71	0	0	0	0	0	0	1.3	30.71
15	Sulfamethoxypyridazine	0	0	0	0	0	0	0	0	0.6	15.74	0.6	15.74
16	Sulfamethazine	0	0	0	0	0	0	0	0	0.6	15.74	0.6	15.74
17	Trimethoprim	0	0	0	0	0	0	0	0	0.3	7.87	0.3	7.87
18	Lincomycin	7.33	29.01	0	0	7.06	29.26	7.01	29.24	7.33	28.88	7.18	29.1
19	Enramycin F	0	0	3.25	13.23	0	0	0	0	0	0	3.25	13.23
	Total	67.66	267.65	55.19	224.78	52.88	219.1	66.8	278.46	67.06	264.21	61.92	250.84
