# Supplementary material for: Excessive use of medically important antimicrobials in food animals in Pakistan: a five-year surveillance survey
Source: Glob Health Action. 2019 Dec 4;12(Suppl):1697541. doi: 10.1080/16549716.2019.1697541 (PMC6896466; doi:10.1080/16549716.2019.1697541)
Supplement: Supplemental Material [file ZGHA_A_1697541_SM6950.pdf]

Table S2: Antimicrobial brands description used between 2013-17						
Brand Count	**Brand Code**	Active Ing-1	**Active Ing-2**	**Active Ing-3**	**Active Ing-4**	**Flocks Treated**
1	**B-101**	Doxycycline 20%	Tylosin 10%	Amantadine 4.5%		3
2	**B-102**	Colistin 4%	Enrofloxacin 10%	Amantadine 4%		1
3	**B-103**	Colistin 48%				6
4	**B-104**	Enrofloxacin 20%				6
5	**B-105**	Colistin 10%	Oxytetracycline 25%	Neomycin 30%		2
6	**B-106**	Colistin 20%				18
7	**B-107**	Colistin 2%	Tylosin 10%	Doxycycline 20%		13
8	**B-108**	Colistin 4%	Enrofloxacin 10%			2
9	**B-109**	Colistin 4%	Enrofloxacin 10%	Amantadine 4%		5
10	**B-110**	Enrofloxacin 10%				25
11	**B-111**	Enrofloxacin 20%				1
12	**B-112**	Amantadine 4%	Enrofloxacin 10%			4
13	**B-113**	Doxycycline 20%	Tylosin 10%	Amantadine 4%		3
14	**B-114**	Norfloxacin 20				1
15	**B-115**	Chlortetracycline 40%	Furaltadone 30%	Neomycin 12%		18
16	**B-116**	Colistin 10%	Oxytetracycline 25%	Neomycin 30%		2
17	**B-117**	Colistin 0.25%	Streptomicin Sul 3.6%	Zn Bacitracin 5.2%	Proc Penicillin 1.2%	1
18	**B-118**	Colistin 20%				3
19	**B-119**	Furaltadone 10%	Tylosin 5%	Erythromycin 10%		1
20	**B-120**	Sulfamethoxypyridazine 5%	Enrofloxacin 7.5%	Sulfamethazine 5%	Trimethoprim 3%	1
21	**B-121**	Colistin 4%	Tylosin 20%	Doxycycline 40%		1
22	**B-122**	Colistin 4%	Tylosin 10%	Doxycycline 20%		1
23	**B-123**	Doxycycline 20%	Tylosin 10%	Amantadine 4%		10
25	**B-124***	Lincomycin				
26	**B-125***	Enramycin				
	***Antimicrobials used as feed additive**					
