# Supplementary material for: Excessive use of medically important antimicrobials in food animals in Pakistan: a five-year surveillance survey
Source: Glob Health Action. 2019 Dec 4;12(Suppl):1697541. doi: 10.1080/16549716.2019.1697541 (PMC6896466; doi:10.1080/16549716.2019.1697541)
Supplement: Supplemental Material [file ZGHA_A_1697541_SM6943.pdf]

Table S1: Farm Records 2013-17														
2013			2014			2015			2016			2017		
Dec-Jan	F25		Dec-Jan	F19		Feb-Mar	F13		Jan-Feb	F7		Jan-Feb	F1	
	B-107*	20 l		B-101	18 kg		B-106	1 l		B-108	3 l		B-104	10 l
	B-110	1 l		B-106	2 l		B-110	5 l		B-110	4 l		B-115	0.5 kg
	B-116	6 kg		B-110	5 l		B-112	18 l		B-112	9 l		B-118	6 l
	B-124	1.232 kg		B-111	0.5 l		B-122	17 l		B-115	1 kg		B-123	16 kg
	Chicks**	19500		B-117	25 kg		B-124	1.2144 kg		B-116	5 kg		B-124	1.276 kg
	Feed***	1400 bags		B-125	0.56 kg		Chicks	19700		B-118	3 l		Chicks	20000
	Weight****	43000 kg		Chicks	19600		Feed	1380 bags		B-123	19 kg		Feed	1450 bags
Feb-Mar	F26			Feed	1400 bags		Weight	41761 kg		B-124	1.188 kg		Weight	46061 kg
	B-107	13 l		Weight	42329 kg	Apr-May	F14			Chicks	20000	Mar-Apr	F2	
	B-109	4 l	Feb-Mar	F20			B-101	33 kg		Feed	1350 bags		B-106	5 l
	B-110	4 l		B-107	13 l		B-106	3 l		Weight	40936 kg		B-110	6 l
	B-115	1 kg		B-109	4 l		B-110	5 l	Mar-Apr	F8			B-115	1 kg
	B-124	1.232 kg		B-110	4 l		B-115	1 kg		B-108	4 l		B-120	12 l
	Chicks	19700		B-115	1 kg		B-124	1.1264 kg		B-115	1 kg		B-123	23.5 kg
	Feed	1400 bags		B-125	0.54 kg		Chicks	20000		B-123	17 kg		B-124	1.188 kg
	Weight	42000 kg		Chicks	20000		Feed	1280 bags		B-124	1.1968 kg		Chicks	20000
Apr-May	F27			Feed	1350 bags		Weight	37862 kg		Chicks	20000		Feed	1350 bags
	B-107	17 l		Weight	40923 kg	Jun-Jul	F15			Feed	1360 bags		Weight	38127 kg
	B-109	7 l	Apr-May	F21			B-106	2 l		Weight	40551 kg	May-Jun	F3	
	B-110	5 l		B-103	2 l		B-107	12 l	May-Jun	F9			B-103	6 l
	B-124	1.173 kg		B-106	2 l		B-110	4 l		B-104	5 l		B-104	10.5 l
	Chicks	19000		B-107	13 l		B-115	1 kg		B-106	20 l		B-115	1 kg
	Feed	1333 bags		B-109	4 l		B-124	1.1616 kg		B-110	4 l		B-123	18.5 kg
	Weight	40000 kg		B-110	5 l		Chicks	20000		B-112	19 l		B-124	1.232 kg
Jun-Jul	F28			B-125	0.52 kg		Feed	1320 bags		B-114	10 l		Chicks	20000
	B-102	8 l		Chicks	20000		Weight	41062 kg		B-123	38 kg		Feed	1400 bags
	B-107	20 l		Feed	1300 bags	Aug-Sep	F16			B-124	1.0384 kg		Weight	41758 kg
	B-110	10 l		Weight	39000 kg		B-106	1 l		Chicks	20000	Jul-Aug	F4	
	B-124	1.2144 kg	Jun-Jul	F22			B-110	3 l		Feed	1180 bags		B-103	5.5 l
	Chicks	20000		B-107	15 l		B-123	12.5 kg		Weight	31353 kg		B-104	7.5 l
	Feed	1380 bags		B-109	4 l		B-124	1.1 kg	Jul-Aug	F10			B-106	2 l
	Weight	42300 kg		B-110	6 l		Chicks	19000		B-104	4 l		B-110	6 l
Aug-Sep	F29			B-125	0.54 kg		Feed	1250 bags		B-106	2 l		B-113	14 kg
	B-103	6 l		Chicks	20000		Weight	37108 kg		B-123	12 kg		B-115	1 kg
	B-106	6 l		Feed	1350 bags	Sep-Oct	F17			B-124	1.1264 kg		B-124	1.144 kg
	B-107	20 l		Weight	40354 kg		B-106	1 l		Chicks	20000		Chicks	20000
	B-110	4 l	Aug-Sep	F23			B-107	18 l		Feed	1280 bags		Feed	1300 bags
	B-115	1.5 kg		B-105	12 kg		B-110	9 l		Weight	39551 kg		Weight	38000 kg
	B-124	1.232 kg		B-106	3 l		B-112	3 l	Sep-Oct	F11		Sep-Oct	F5	
	Chicks	20000		B-107	15 l		B-124	1.254 kg		B-106	2 l		B-103	2 l
	Feed	1400 bags		B-110	8 l		Chicks	20000		B-110	5 l		B-104	7 l
	Weight	42500 kg		B-115	1.5 kg		Feed	1425 bags		B-119	20 kg		B-113	15 kg
Oct-Nov	F30			B-125	0.528 kg		Weight	44046 kg		B-123	3 kg		B-115	1 kg
	B-103	8 l		Chicks	20000	Nov-Dec	F18			B-124	1.232 kg		B-124	1.276 kg
	B-106	6 l		Feed	1320 bags		B-101	11 kg		Chicks	20000		Chicks	19500
	B-110	6 l		Weight	40352 kg		B-110	4 l		Feed	1400 bags		Feed	1450 bags
	B-115	1 kg	Oct-Nov	F24			B-115	1.5 kg		Weight	43101 kg		Weight	45106 kg
	B-121	13 kg		B-105	2 kg		B-118	3 l	Nov-Dec	F12		Nov-Dec	F6	
	B-124	1.2496 kg		B-106	2 l		B-124	1.2056 kg		B-106	2 l		B-106	3 l
	Chicks	20000		B-107	13 l		Chicks	20000		B-107	5 l		B-110	6 l
	Feed	1420 bags		B-110	6 l		Feed	1370 bags		B-110	4 l		B-113	14 kg
	Weight	43000 kg		B-115	1 kg		Weight	39517 kg		B-115	1 kg		B-115	1 kg
				B-125	0.56 kg					B-123	3 kg		B-124	1.2144 kg
				Chicks	19800					B-124	1.232 kg		Chicks	19500
				Feed	1400 bags					Chicks	20000		Feed	1380 bags
				Weight	42569 kg					Feed	1400 bags		Weight	44766 kg
										Weight	44411 kg			
	*Anonymised brand name													
	**Number of chicks placed at farm													
	***Feed bags; 1 bag = 50 kg													
	****Final Flock Weight													
